# Comparative Accuracy of Colonoscopy, Computed Tomography (CT), and Magnetic Resonance Imaging (MRI) for the Preoperative Localization of Colorectal Cancer: A Prospective Cohort Study

**DOI:** 10.7759/cureus.95621

**Published:** 2025-10-28

**Authors:** Aikaterini Sarafi, Aikaterini Leventi, Dionysios Katsaounis, Klaoudia Athitaki, Maria Igoumenidi, Nikolaos P Tasis, Theodoros Tsirlis, Iraklis Katsoulis, Dimitris P Korkolis

**Affiliations:** 1 Surgical Oncology, General Anticancer and Oncology Hospital of Athens "Saint Savvas", Athens, GRC; 2 General Surgery, General Hospital of Athens "Elpis", Athens, GRC

**Keywords:** colonoscopy, colorectal cancer (crc), ct, localization, pelvic mri

## Abstract

Background and objectives: With the increasing use of laparoscopic and robotic approaches, accurate preoperative localization of colorectal cancer (CRC) is crucial for surgical planning. The present study aimed to compare the accuracy of colonoscopy, computed tomography (CT), and magnetic resonance imaging (MRI) for preoperative tumor localization in patients diagnosed with CRC.

Methods: This single-center prospective study evaluated the accuracy of colonoscopy, CT, and MRI for rectal cancer as tools for preoperative tumor localization. Information from preoperative investigations was recorded prospectively and compared with intraoperative findings. All colonic segments were included.

Results: Colonoscopy was the most accurate method, with an overall accuracy of 82.4%. However, it was less effective in the hepatic and splenic flexures, with failure rates of 88% and 100%, respectively. CT demonstrated an overall accuracy of 57%, which improved to 74% for visible lesions. MRI was highly accurate for localizing rectal cancers.

Conclusions: Colonoscopy remains the gold standard for preoperative tumor localization despite its limitations in specific colonic segments. CT offers complementary information, but it was less accurate overall. MRI was a valuable tool for detecting rectal cancer. Future research should focus on improving localization methods, particularly in anatomically challenging areas.

## Introduction

Accurate preoperative localization of colorectal cancer (CRC) is essential for surgical planning, ensuring that the appropriate colonic segment is resected according to oncological principles. This is particularly critical in laparoscopic procedures, where intraoperative palpation is limited. Mislocalization can lead to incorrect trocar placement and suboptimal operative setup, including improper positioning of the patient and arrangement of surgical instruments [1]. These issues can complicate access to the tumor and may require repositioning or adjustment during surgery. Furthermore, mislocalization may result in the resection of the wrong colonic segment or an unnecessarily extensive resection, increasing operative time and the risk of complications. In some cases, failure to localize the tumor intraoperatively necessitates conversion to open surgery, leading to higher patient morbidity. Therefore, precise preoperative localization plays a key role in optimizing surgical setup, minimizing intraoperative complications, and improving outcomes.

Although colonoscopy is considered the gold standard method for the detection of CRC, its reported accuracy for tumor localization varies in the literature, due in part to the absence of reliable anatomical landmarks between the anal verge and the ileocecal valve [2-5]. In addition, preoperative computed tomography (CT), traditionally used for staging, can also aid in operative planning by allowing the visualization of the tumor and its relationships with other anatomical landmarks. The present study aimed to examine the accuracy of colonoscopy and CT as tools for tumor localization compared with intraoperative findings.

## Materials and methods

Study design

This study involved a retrospective analysis of anonymized clinical data collected prospectively from patients admitted to the Department of Surgical Oncology of General Anticancer and Oncology Hospital of Athens "Saint Savvas" in Athens, Greece, from January 1, 2023, until January 1, 2024. All patients had previously signed informed consent forms that permitted the use of their data for research purposes. The Institutional Review Board reviewed the study and granted a waiver of formal IRB approval (11061/17-07-2025), as the research posed minimal risk, utilized deidentified data, and was conducted in the context of an internal audit.

In accordance with standard clinical practice, all patients underwent colonoscopy and CT prior to admission to identify and stage the tumor. In addition, for tumors located below the rectosigmoid junction, pelvic magnetic resonance imaging (MRI) with a rectal protocol was performed. Colonoscopy was performed by an expert endoscopist who specified the site of the colon affected by the lesion, and CT and MRI were performed by an expert radiologist who was blinded to each other's comments. Intraoperatively, the surgeon and assistant agreed on the appropriate tumor location, and their findings were recorded in Table 1.

**Table 1:** Colon segmentation criteria

Segment no.	Segment name	Anatomical boundaries/description
1	Right colon	From the ileocecal valve to 10 cm proximal to the hepatic flexure
2	Hepatic flexure	10 cm before and after the hepatic curve
3	Transverse colon	From 10 cm after the hepatic flexure to 10 cm before the splenic flexure
4	Splenic flexure	10 cm before and after the splenic curve
5	Descending colon	From the lowest retroperitoneal attachment to 10 cm before the splenic curve
6	Sigmoid colon	From the descending colon's retroperitoneal end to the peritoneal reflection
7	Rectum	Between the sigmoid colon and anorectal junction; marked by taenia coli coalescence and loss of epiploic appendages

All procedures were performed using a flexible endoscope. However, bowel preparation protocols were not entirely uniform across patients, as they were based on standard clinical practice at the time. Adequate bowel preparation was confirmed in all cases, defined as a Boston Bowel Preparation Score (BBPS) of ≥6, with no individual segment scoring less than 2. The placement of the tattoo followed a standardized protocol in our study. Specifically, tattoos were placed endoscopically distal to the tumor site, typically 2-3 cm beyond the lesion, to ensure accurate intraoperative localization. The criteria used for colon segmentation based on colonoscopic findings are summarized in Table 2.

**Table 2:** Colonoscopy-based criteria for colonic segment classification

Colon segment	Endoscopic landmarks/criteria
Cecum	Identification of the appendiceal orifice and ileocecal valve; presence of a wide lumen
Ascending colon	Between the cecum and hepatic flexure; presence of longitudinal folds
Hepatic flexure	Sharp bend near the liver; visible as an angulation on colonoscopy
Transverse colon	Segment between the hepatic and splenic flexures; typically more mobile with haustral folds
Splenic flexure	Sharp bend near the spleen; often difficult to visualize completely
Descending colon	From the splenic flexure to the sigmoid colon; straighter course, relatively fixed
Sigmoid colon	Tortuous S-shaped curve distal to the descending colon
Rectum	Distal straight segment with the absence of haustra; visible landmarks like the rectosigmoid junction

Patient data were prospectively recorded in anonymized and standardized forms for the time period of the study, and researchers were blinded to other information except for those recorded in the forms. Forms included preoperative information such as age, sex, body mass index (BMI), preoperative diagnosis and histology, type of neoadjuvant treatment, localization of the tumor by colonoscopy, use of a tattoo, and visibility and localization of the lesion from preoperative CT and MRI in rectal lesions. Intraoperative information included the type of procedure (open or laparoscopic) and the need to modify the initial approach due to incorrect tumor localization, the actual location of the tumor, and the histopathological type, stage, and lesion diameter (cm) from the pathology report.

**Figure 1:**
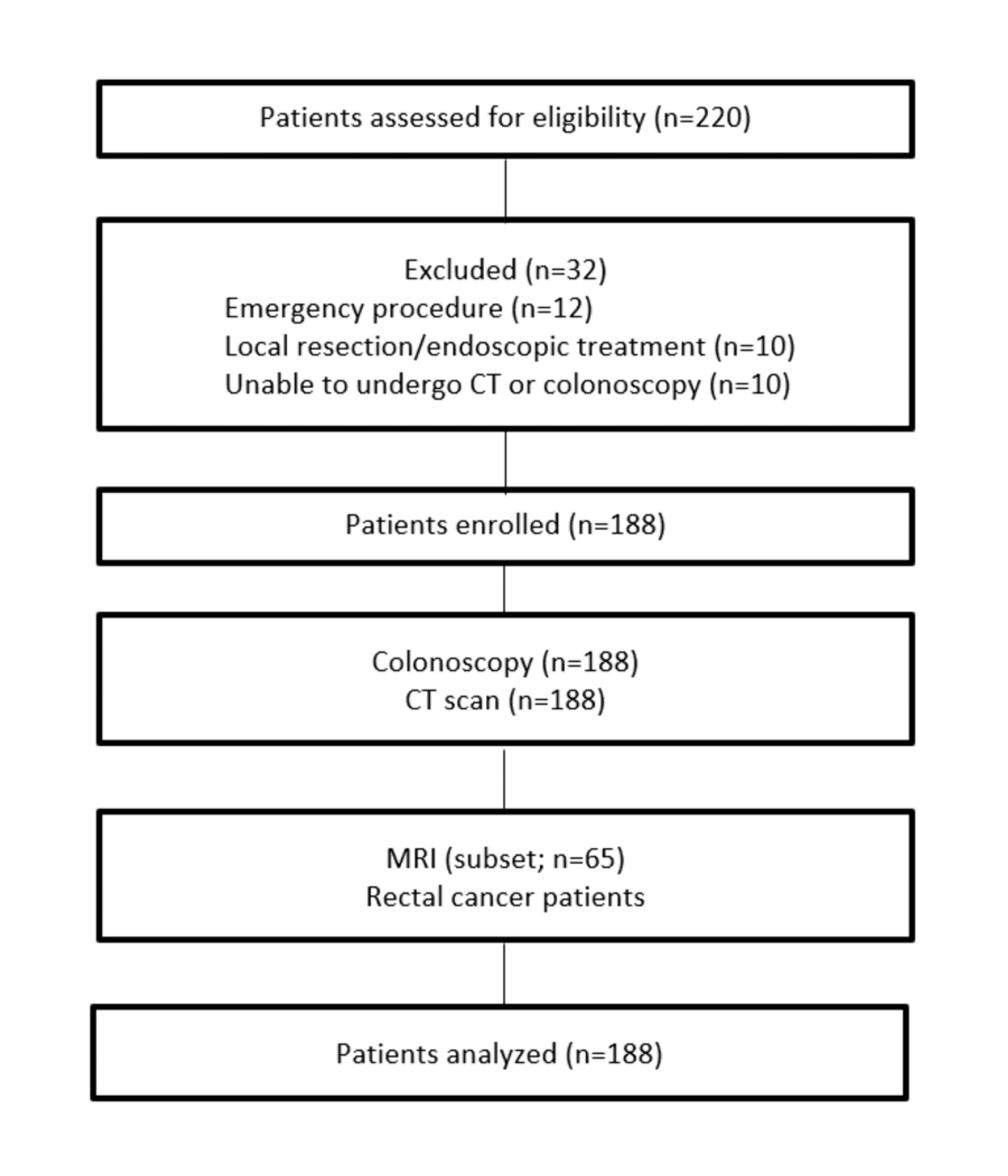
STROBE flowchart The flowchart illustrates the inclusion and exclusion of patients admitted to the Department of Surgical Oncology from January 1, 2023, to January 1, 2024. A total of 220 patients were initially assessed for eligibility. Of these, 32 patients were excluded due to reasons including inability to undergo preoperative CT or colonoscopy, emergency procedures, local resection, or curative endoscopic treatment. The final study cohort consisted of 188 patients who met all eligibility criteria and underwent elective surgical resection. STROBE: Strengthening the Reporting of Observational Studies in Epidemiology; CT: computed tomography; MRI: magnetic resonance imaging

The study included all patients admitted to the department in the abovementioned period, who had a histologically confirmed diagnosis of dysplastic adenoma or adenocarcinoma of the colon and rectum and were scheduled for elective surgical resection. The exclusion criteria were the inability to undergo preoperative CT or colonoscopy, emergency procedures, local resection, and curative endoscopic treatment. A detailed flowchart outlining patient inclusion and exclusion is presented in Figure 1, in accordance with the Strengthening the Reporting of Observational Studies in Epidemiology (STROBE) guidelines. 

This study was based on real-world data from patients referred to our institution. Imaging and endoscopic reports were obtained from clinical files and originated from multiple public and private diagnostic centers. As such, blinding of operators was not feasible, and standardization of interpretation or assessment of interobserver variability could not be performed. The findings reflect routine clinical practice and the heterogeneity inherent in real-world diagnostic workflows.

The primary outcome was the accuracy of preoperative tumor localization, defined as the concordance between the location identified by colonoscopy, CT, and MRI (for rectal cancers) and the intraoperative tumor location, which served as the reference standard. Accuracy was assessed both overall and stratified by tumor location (e.g., right colon, left colon, rectum) to evaluate modality performance across anatomical regions. The secondary outcome was the proportion of cases in which tumor mislocalization led to a change in the planned operative procedure.

Statistical analysis

Data are expressed as means±standard deviations (SDs) for continuous variables and as frequencies and percentages for categorical variables. Agreement between colonoscopy, abdominal CT, and MRI with the gold standard (tumor location during surgery) was evaluated via linear-weighted Cohen's kappa values according to the guidelines of Landis and Koch [6]. All the statistical tests were two-sided, and differences with p<0.05 were considered statistically significant. All analyses were performed using IBM SPSS Statistics for Windows Version 21.0 (IBM Corp., Armonk, New York, United States) and MedCalc Version 20 (MedCalc Software Ltd., Ostend, Belgium). Kappa values reflecting agreement were graded as follows: 0.01-0.2, slight; 0.21-0.40, fair; 0.41-0.60, moderate; 0.61-0.80, substantial; and 0.81-1, almost perfect or perfect.

## Results

Among the 188 patients enrolled, 103 (54.8%) were male, the mean age was 68.93±10.66 years, and the mean BMI was 26.42±4.33 kg/m². The maximum lesion diameter (dmax) was reported in 169 patients, with a mean dmax of 3.33±1.72 cm. Almost one-quarter of patients (21.3%; n=40) underwent a laparoscopic procedure.

With respect to pathological data, lesion staging was distributed as follows: T1, 15 (7.9%); T2, 52 (27.6%); T3, 86 (45.7%); T4, 15 (7.9%); and high-grade adenoma or in situ adenocarcinoma, 10 (5.3%). A complete pathological response after neoadjuvant treatment was observed in 10 (5.3%) patients, although the T stage could not be determined. The results of the demographic and pathological analyses are summarized in Table 3.

**Table 3:** Demographic and pathologic characteristics of the sample Demographic (age, gender, BMI) and pathological data (tumor stage, histopathology, neoadjuvant response, diameter of the lesion) for 188 patients BMI: body mass index; TRG: tumor regression grade

Characteristics		No. of patients (%)
Sex (%)		
Male (%)		103 (54.8%)
Female (%)		85 (45.2%)
Age (years) (mean±SD)		68.93±10.66
BMI (kg/m^2^) (mean±SD)		26.42±4.33
Diameter of the lesion (cm) (mean±SD)		3.33±1.72 cm
Neoadjuvant treatment (%) *only for rectal cancer		53/68 (78%)
T stage (%)		
T1	15 (7.9%)	
T2	52 (27.6%)	
T3	86 (45.7%)	
T4	15 (7.9%)	
TRG 0	10 (5.3%)	
High-grade adenoma or in situ adenocarcinoma	10 (5.3%)	

Localization via colonoscopy

According to the colonoscopy reports, most lesions were located in the rectum (34.6%; n=65), followed by the sigmoid (29.8%; n=56) and right colon (22.3%; n=42). Tattoos were placed in 33 (17.6%) patients.

**Figure 2:**
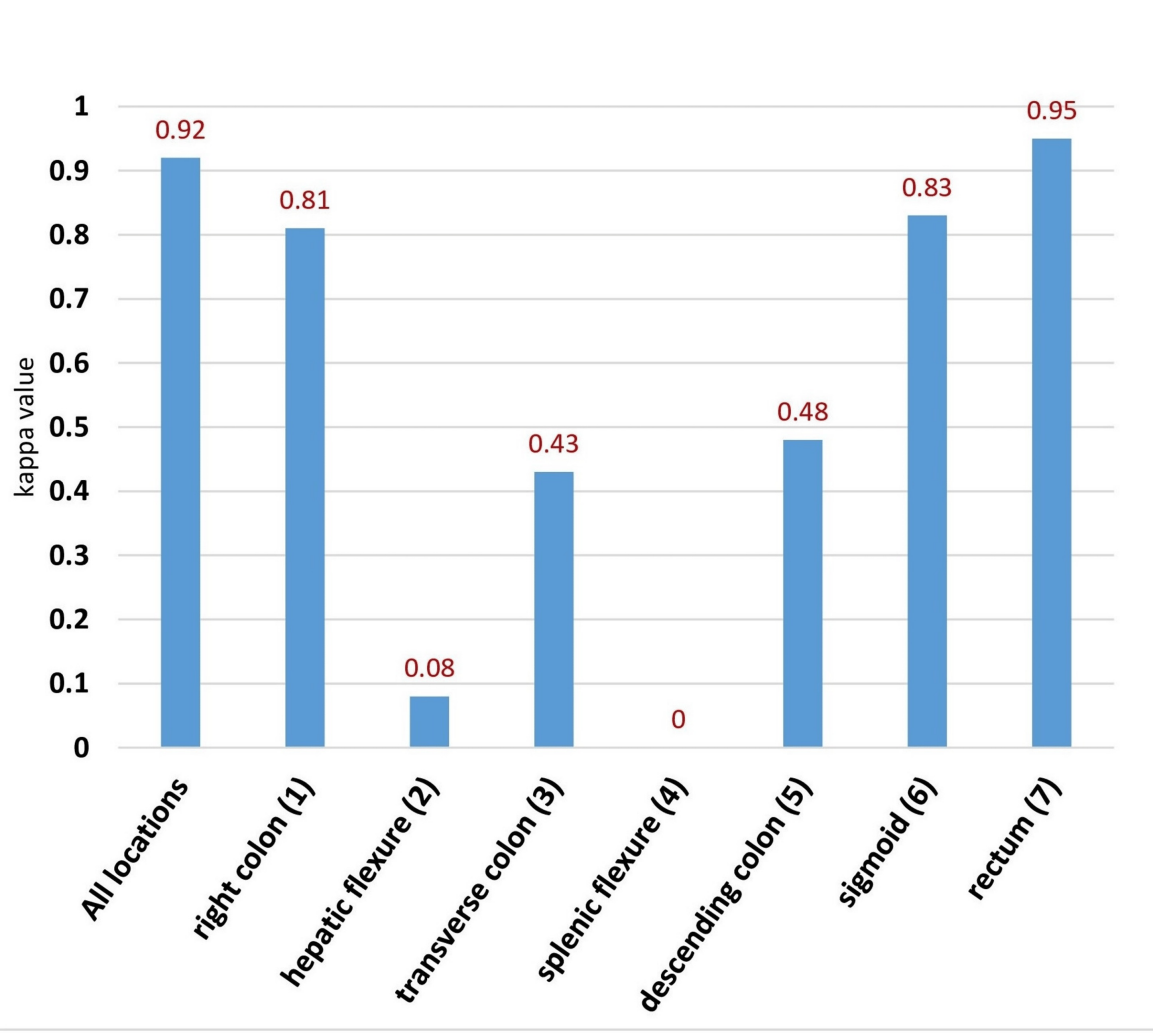
Agreement between colonoscopy and intraoperative tumor localization The x-axis represents the different colonic segments, while the y-axis indicates the kappa value. There was a strong agreement between intraoperative findings and endoscopic localizations, as indicated by the high kappa value. Endoscopic mislocalization was more common in the hepatic and splenic flexures.

When intraoperative findings were compared with those from colonoscopy, perfect agreement was observed, with a kappa value of 0.92 (95% CI: 0.89-0.95; p<0.005). Correct localization was observed in 155 (82.4%) patients. Subgroup analysis revealed that lesions in the rectum (0.95; 95% CI: 0.91-0.99; p<0.005), sigmoid (0.83; 95% CI: 0.74-0.92; p<0.005), and right colon (0.81; 95% CI: 0.70-0.90; p<0.005) were in perfect agreement. Endoscopic mislocalization was more frequently observed in the hepatic flexure (kappa value=0.08) and splenic flexure (kappa value=0.00), where almost no lesions were accurately localized, and the threshold for statistical significance was not reached. The results of endoscopic localization are summarized in Figure 2.

The agreement between colonoscopy and operative localization for the subgroup of patients who had a tattoo placed during colonoscopy, using the linear-weighted kappa (95% CI), revealed no improvement in accuracy between the group with tattoo injection and those without (kappa value=0.70 versus kappa value=0.90, respectively).

Localization via abdominal CT

Preoperative CT revealed colonic lesions in 144 (76.6%) patients, with correct localization reported in 107 (74.3%) patients. Most lesions were reported to be located in the sigmoid (29.8%; n=56) and rectum (18.6%; n=35). There was moderate agreement between abdominal CT and operative localization, with a linear-weighted kappa of 0.55 (95% CI: 0.41-0.66; p<0.005). Sub-analysis indicated that only lesions in the right colon (0.80; 95% CI: 0.70-0.91; p<0.005) yielded perfect agreement.

**Figure 3:**
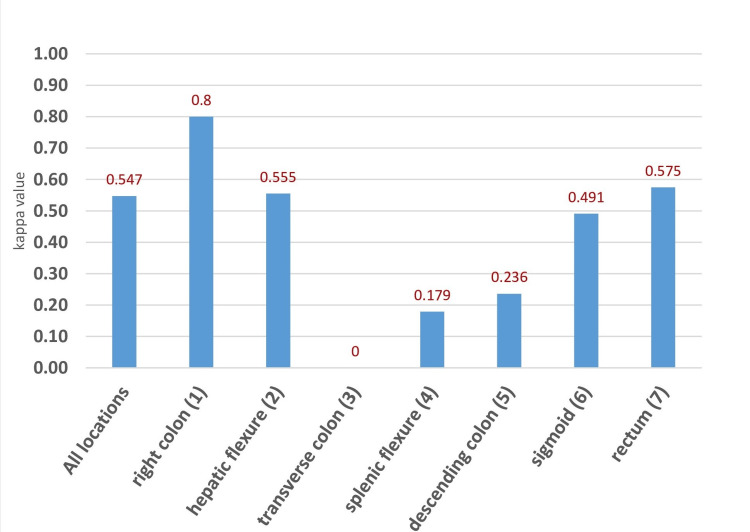
Agreement between CΤ scan and intraoperative tumor localization Accuracy of preoperative CT in localizing colonic lesions in 144 cases. The x-axis represents the different colonic segments, while the y-axis indicates the kappa value. Lesions in the right colon presented perfect agreement. CT mislocalization was more frequently observed in the transverse colon, splenic flexure, and descending colon. CT: computed tomography

In terms of tumor location, mislocalization was more frequently observed in the transverse colon (100%), splenic flexure (83%), and descending colon (71%); however, these differences were not statistically significant. The results of CT-based localization are summarized in Figure 3.

With respect to the percentage of nonvisible lesions on CT (23.4%; n=24), this subset was analyzed according to diameter, with most lesions (72%; n=32) exhibiting a diameter >2 cm.

Localization via MRI

All patients with rectal cancer underwent pelvic MRI according to the protocol. A subgroup of cases identified by endoscopy as rectal cancers (n=65) was examined to evaluate the accuracy of MRI in tumor localization. Sixty-three patients underwent rectal MRI, and in two (3%) patients, MRI was not able to identify the lesion. Among the remaining 61 cases, 60 were accurately located when compared with intraoperative localization.

MRI yielded a near-perfect agreement, with a linear-weighted kappa of 0.87 (95% CI: 0.79-0.95; p<0.005).

Data from surgery

Laparoscopic surgery was performed in 21.3% (n=40) of patients, whereas open surgery was performed in the majority (78.7%; n=148).

The need for a change in the initial surgical plan due to incorrect localization, from either colonoscopy or imaging, occurred in 20 (10%) patients. Six (15%) patients required conversion from a minimally invasive procedure to open surgery. In all of these patients, the initial surgical plan was laparoscopic anterior resection; however, intraoperatively, the lesion was found to be a low-lying rectal cancer. In four patients, the localization error differed by two colonic segments (sigmoid to splenic flexure and ascending to transverse), and in the remaining 16 patients, the error differed by one colonic segment. The intraoperative data are summarized in Table 4.

**Table 4:** Intraoperative changes This table presents the modification of the initial surgical approach in concordance with the intraoperative findings. In 20 cases, the initial surgical plan was altered due to incorrect localization. In six cases, we had to convert from minimal invasive to open surgery. Localization errors varied by one or two colonic segments in the majority of cases.

Initial plan	Modified		Reason	No cases (%) modified/initial
Laparoscopic resection	Convert to open		Low-lying rectal cancer, technical difficulties	6/40 (15%)
Sigmoidectomy	Left colectomy		Descending colon	2/6 (33.3%)
Extended right colectomy	Right colectomy		Ascending colon	3/34 (8.8%)
Left hemicolectomy	Extended right colectomy		Middle transverse colon	3/4 (75%)
			Hepatic flexure	1/7 (14.3%)
Left hemicolectomy		Subtotal colectomy	Splenic flexure	5/5 (100%)

## Discussion

This prospective observational single-center study compared colonoscopy, CT, and MRI for preoperative tumor localization in patients with CRC. Accurate localization is critical for surgical planning, especially with minimally invasive approaches such as laparoscopy and robotics, where errors can result in inappropriate trocar placement, misidentification of the target segment, and conversion to open surgery [7].

In this study, we evaluated how colonoscopy, CT, and MRI findings correlated with intraoperative localization across all colonic segments. Our findings support the notion that colonoscopy, despite its variability, remains the gold standard, as in our series, colonoscopy demonstrated an overall accuracy of 82.4%, with a striking 98% accuracy for rectal lesions. However, its performance dropped sharply at anatomical flexures, particularly the hepatic and splenic flexures, where mislocalization rates reached 88% and 100%, respectively, echoing the challenges described in prior studies [8,9] with notably low accuracy, especially around the transverse colon [8]. These findings reinforce the challenges of endoscopic navigation in anatomically complex regions such as the flexural areas.

More advanced imaging modalities, like virtual colonoscopy (CT colonography), could offer a non-invasive alternative with promising accuracy, especially in polyp detection, but its role in precise tumor localization remains limited in routine preoperative planning. Studies such as Fenlon et al. [10] and the National CT Colonography Trial [11] suggest that while virtual colonoscopy is accurate for screening, it may not consistently outperform conventional colonoscopy in surgical localization.

Although endoscopic tattooing was used in many cases, we found no correlation between tattoo use and improved localization accuracy by the endoscopist. Despite a systematic review and meta-analysis by Acuna et al. [12], which concluded that colonoscopic tattooing reduces localization errors, our findings do not support this. This can be explained by the fact that while the tattoo serves primarily as an intraoperative visual aid for the surgeon, it does not necessarily improve the endoscopist's ability to accurately identify the specific colonic segment involved preoperatively.

In our study, CT localization demonstrated lower accuracy, with correct identification in only 57% of all lesions, rising to 74% when only visible tumors were considered. Localization was most accurate for right-sided lesions and least accurate for the transverse colon, splenic flexure, and descending colon. Notably, lesion visibility did not consistently correlate with size; even tumors >2 cm were occasionally missed, likely due to the collapsed lumen or poor contrast differentiation in the unprepped colon.

Previous studies have reported similarly mixed results regarding the performance of CT for tumor localization. In a retrospective analysis of 104 patients with colon cancer, Lee et al. [3] compared the localization accuracy of colonoscopy and CT against surgical findings. Consistent with our results, colonoscopy had an accuracy of 79.8%, while CT was accurate in only 50% of cases and failed to detect tumors in 32.7% of patients. Similarly, in a multicenter study involving 364 patients with colorectal lesions, Moug et al. [13] reported preoperative accuracy rates of 82% for colonoscopy and 59% for CT. However, when only visible lesions on CT were considered, the accuracy improved to 80%.

In contrast, Feuerlein et al. [14], using intraoperative findings as the gold standard, concluded that preoperative CT was more accurate than colonoscopy for localizing colonic tumors. Notably, this study excluded patients with rectal and cecal cancers, and CT accuracy increased from 40% to 80% when the radiologist was informed of the tumor location in advance.

More recently, the first prospective multicenter study on this topic, the PLoCoS Study, by Manigrasso et al. [15] assessed the accuracy of CT and colonoscopy in identifying the location of colonic lesions compared with surgical findings. Among 729 patients, colonoscopy and CT achieved accuracy rates of 74.6% and 70%, respectively. In that study, and in line with our own findings, but contrary to Solon et al. [8], who reported lower CT accuracy in the right colon, colonoscopy failed to localize tumors accurately in the descending colon.

Overall, the variability in CT performance, particularly in localizing tumors in the left-sided or transverse colon, has been attributed to differences in bowel preparation, tumor size, and radiologist interpretation protocols [8,14].

In contrast, MRI proved highly reliable for rectal tumor localization, matching intraoperative findings in 98% of cases. This is consistent with previous work [16] and highlights MRI's diagnostic superiority in differentiating rectal from sigmoid tumors. Emerging radiomic approaches further enhance MRI precision and offer promise for preoperative planning, especially for individualized therapeutic strategies [17].

One of the most clinically relevant findings was that 10.6% of patients required changes in the surgical plan due to incorrect preoperative localization. This reflects trends reported in other cohorts, reinforcing that localization failure in anatomically ambiguous regions can directly impact operative decision-making [18]. These changes included trocar repositioning, intraoperative re-scoping, or conversion to open surgery. Most occurred in the transverse colon and splenic flexure, areas where colonoscopy and CT were both least reliable. Given the surgical ambiguity of flexural tumors, which may require extended or segmental resection, preoperative mislocalization can significantly affect oncological outcomes and operative efficiency [19].

To this well-recognized problem, practical solutions that could improve localization include facilities like intraoperative endoscopy, which enables direct lesion identification when preoperative imaging is inconclusive, and artificial intelligence (AI)-assisted imaging interpretation, including radiomics, which may reduce variability in radiologist and endoscopist reporting. Though these approaches are not readily available in all institutions, simple measures like standardized reporting templates, such as structured radiology checklists that enforce anatomical labeling, and novel tattooing methods, such as autologous blood or radiopaque markers, which may offer safer or more consistent intraoperative detection [20,21], could also be helpful.

The current trend in research is oriented toward the development of AI imaging integration (widely known as radiomics). The latter has demonstrated improved consistency in tumor localization, particularly for complex or radiologically obscure lesions [17,22]. This is particularly important, as surgical outcomes appear to be influenced more by localization precision than by the extent of resection, with minimally invasive approaches showing favorable results even in difficult-to-localize tumors [23].

This study has several limitations. The single-center design and relatively small sample size may limit the generalizability of our findings, particularly for less common tumor locations. Additionally, the inability to retrospectively review non-visible CT lesions represents a missed opportunity for further diagnostic refinement and should be addressed in future protocols. We also acknowledge the absence of multicenter validation, limited sample diversity, and the potential for observer bias in imaging interpretation. However, the prospective nature of our data collection and the use of intraoperative findings as the gold standard for tumor localization enhance the reliability of the results. Importantly, this study reflects a real-world, pragmatic approach embedded in routine clinical workflows. As such, the data provide valuable insights into everyday surgical planning and decision-making, underscoring the practical relevance and applicability of our findings despite these limitations.

In conclusion, colonoscopy remains the cornerstone of preoperative localization, particularly when supported by tattooing or intraoperative endoscopic assessment. CT provides complementary structural information but falls short in precise localization. MRI, especially for rectal lesions, is highly accurate and should be considered a standard component in multidisciplinary planning. Continued exploration of advanced imaging techniques and AI-enhanced analysis may further reduce localization errors and optimize patient outcomes.

## Conclusions

Our findings support the use of colonoscopy as the standard reference modality for preoperative tumor localization in CRC, given its overall high accuracy and direct visualization of lesions. However, clinicians should exercise caution in interpreting colonoscopic localization in anatomically complex regions such as the hepatic and splenic flexures, where mislocalization rates are significantly higher. MRI should be considered essential for rectal cancer, as it consistently demonstrates near-perfect accuracy and provides crucial anatomical detail for surgical planning. In contrast, CT alone is insufficient for reliable preoperative localization, particularly in left-sided and transverse colonic lesions. While CT may have some utility in detecting right-sided tumors and offering complementary anatomical context, its variable accuracy and frequent non-visualization of lesions limit its standalone value. Given that preoperative mislocalization led to changes in surgical strategy in over 10% of cases, particularly in flexural tumors, accurate preoperative localization has direct clinical implications. To enhance surgical planning and minimize intraoperative adjustments or conversions, future research should prioritize the development and validation of AI-assisted imaging tools, radiomics, and intraoperative adjuncts that can overcome the current limitations of standard modalities.
